# Ozone major blood ozonation in the management of dermatomyositis: a case report

**DOI:** 10.3389/fimmu.2026.1767266

**Published:** 2026-06-15

**Authors:** Yunbo Xie, Xinyu Cui, Jiangang Liu, Guohua Song

**Affiliations:** 1 Ozone Biomedical Research Institute, School of Clinical and Basic Medical Sciences of Shandong First Medical University & Shandong Academy of Medical Science, Jinan, China; 2 Shandong Jiangang Hospital Management Co., Ltd., Huaiyin Jiangang Hospital, Jinan, China

**Keywords:** autoimmune disease (AD), dermatomyositis, immunomodulation, ozone major blood ozonation, skin ulcer

## Abstract

**Objective:**

This case report describes the potential clinical benefit and safety of ozone major blood ozonation (O_3_-MBO) in the treatment of glucocorticoid (GC)-dependent dermatomyositis.

**Methods:**

The patient received an initial 15-day daily course of O_3_-MBO (at a concentration of 80 μg/mL), followed by repeated cycles of 15-day treatment alternating with 15-day rest periods over approximately 15 months. Laboratory parameters and cutaneous lesion status were continuously monitored during the treatment period.

**Results:**

In the case presented, the patient added 15 days of O_3_-MBO therapy and 15 days of rest as adjunctive therapy without increasing the hormone dosage, which was associated with reductions in serum levels of CK, AST, and ALT, healed the skin ulcers on the shoulders, normalized the skin, reduced the facial edema, and was associated with reduced corticosteroid-related side effects.

**Conclusion:**

O_3_-MBO may serve as a valuable adjunctive treatment for patients with corticosteroid dependence or refractory dermatological symptoms.

## Introduction

Dermatomyositis is an autoimmune disease characterized by skin lesions and muscle inflammation (1). Although traditional treatment methods such as corticosteroids and immunosuppressants may be effective, some hormone-dependent patients may experience recurrent symptoms, and high-dose use of hormones can lead to serious complications.

Ozone, first introduced for disinfection purposes during World War I (2), has more recently been studied for its therapeutic potential. Research has shown that autohemotherapy using appropriate concentrations of ozone is both safe and effective (3, 4). At these concentrations, ozone induces a mild, transient, and controlled oxidative stress, which stimulates the synthesis of antioxidant enzymes (5) and reduces endogenous oxidative stress (6, 7). During the wound healing process, ozonation can also stimulate the production of growth factors, such as vascular endothelial growth factor (VEGF), platelet-derived growth factor (PDGF), and transforming growth factor beta (TGF-β). Previous experimental studies have demonstrated that ozone therapy may reduce TNF-α production and exert immunomodulatory effects through regulation of inflammatory signaling pathways (8, 9).

In recent years, advances in ozone delivery technologies—including wearable and controlled ozone-generating systems—have enabled more precise and localized therapeutic applications, particularly in dermatological conditions (10–13). These systems have demonstrated promising possible therapeutic role in infected wounds and chronic ulcers, with favorable safety and biocompatibility profiles. Despite these developments, the application of systemic O_3_-MBO in autoimmune diseases such as dermatomyositis remains poorly characterized.

This case study documents for the first time the application of O_3_-MBO as a supplementary treatment for dermatomyositis-associated skin lesions. The therapeutic intervention was associated with cutaneous healing and biochemical improvement, without observed adverse effects. This outcome underlines the potential of O_3_-MBO as a safe and efficacious alternative approach for the management of dermatomyositis-related cutaneous symptoms, warranting further investigation and consideration in clinical practice.

## Disease description

**Figure 1:**
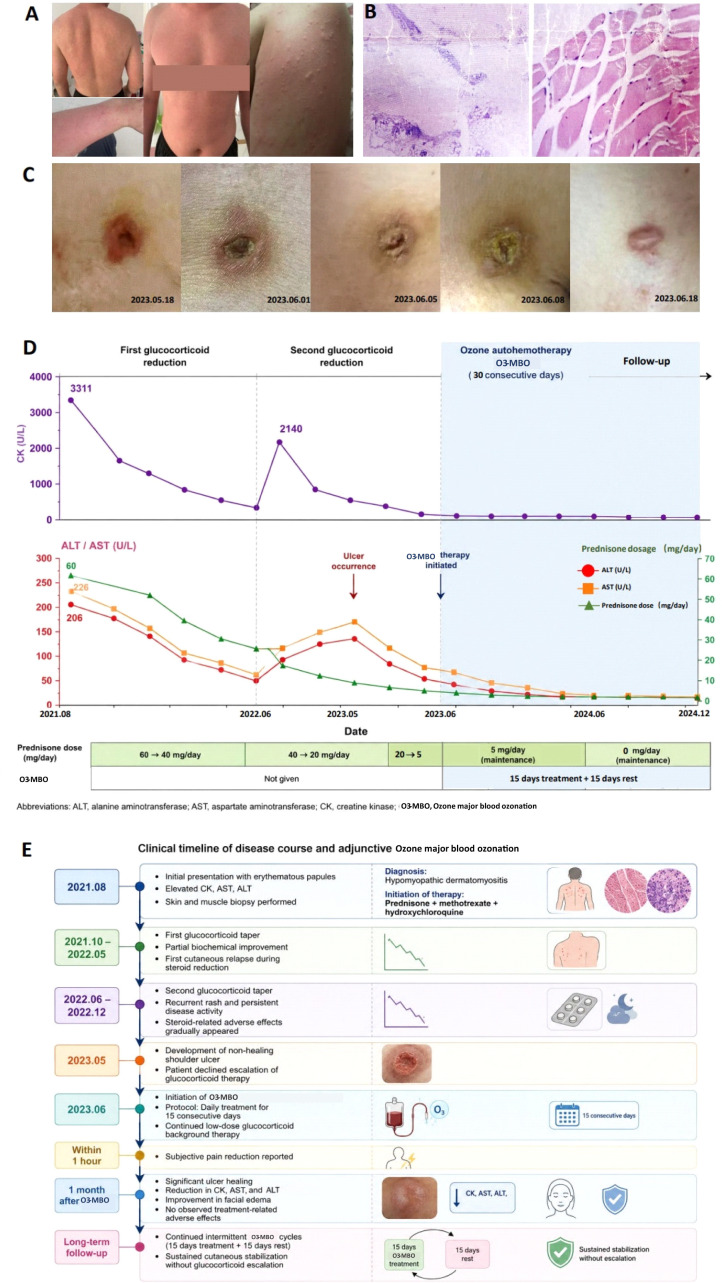
Clinical course and treatment response. **(A)** Erythematous papules visible on the skin at initial presentation. **(B)** Histopathological examination of a muscle biopsy specimen from the left lower limb revealed localized edema and loss of striations (hematoxylin and eosin staining; original magnification ×200; scale bar = 50 μm). **(C)** Progression of shoulder ulcers before and after initiation of autologous ozone therapy. Images were taken on days 1, 15, 19, 22, and 32 after initiation of O_3_-MBO therapy, under standardized lighting and photographic distance to ensure comparability across time points. **(D)** Longitudinal changes in laboratory parameters (CK, AST, ALT) and glucocorticoid use. Shaded areas indicate the period of O_3_-MBO. Reference upper limits of normal: CK ≤ 200 U/L; AST ≤ 40 U/L; ALT ≤ 40 U/L. The y-axis on the left displays enzyme concentrations; the y-axis on the right displays prednisone dose (mg/day). **(E)** Summary of the clinical timeline of disease progression and adjunctive O_3_-MBO therapy, with month 0 corresponding to the date of initial diagnosis. Key clinical events—including glucocorticoid tapering, recurrent rash, ulcer onset, O_3_-MBO initiation, and longitudinal clinical response—are annotated. ALT, alanine aminotransferase; AST, aspartate aminotransferase; CK, creatine kinase; H&E, hematoxylin and eosin.

A 30-year-old male (now 32 years old at the time of consent for publication) presented at the outpatient clinic with a one-month history of erythematous papules (**Figure 1A**). The patient has no history of hypertension, diabetes mellitus, coronary artery disease, or allergies to food or medications. There is also no history of tobacco or alcohol use, and no familial history of similar conditions.

Hepatic impairment markers were substantially elevated, with an alanine aminotransferase concentration of 206 U/L and an aspartate aminotransferase concentration of 226 U/L. Muscle enzyme levels were also substantially elevated, with a creatine kinase concentration of 3311 U/L, a lactate dehydrogenase concentration of 571 U/L, and a creatine kinase myocardial band concentration of 78 ng/mL (CK-MB measured as mass concentration; reference upper limit: 5.0 ng/mL). The electrocardiogram revealed a sinus rhythm with no abnormalities in the ST segment. Although a comprehensive immunologic examination shows a positive antinuclear (ANA) antibody, result, while other autoimmune markers were not diagnostic of a specific connective tissue disease. Myositis-specific antibodies (including Mi-2, MDA5, TIF1-γ, and NXP2) were not assessed at the time of diagnosis, representing a limitation. Electromyography showed no significant abnormalities. However, muscle biopsy of the left lower extremity revealed localized edema and loss of transverse striations (**Figure 1B**). Skin biopsy demonstrated mild epidermal hyperkeratosis, focal basal cell liquefaction, and perivascular mononuclear infiltration. Despite the absence of clinically significant muscle weakness, the combination of elevated muscle enzymes and histopathological findings supported subclinical muscle involvement.

The diagnosis of dermatomyositis was established by the multidisciplinary team based on the combination of characteristic cutaneous findings, elevated muscle enzymes, and supportive muscle biopsy showing localized edema and loss of striations, following the classical Bohan and Peter criteria. Although the patient did not exhibit all of the specific cutaneous features required for high-confidence classification under the 2017 EULAR/ACR criteria (e.g., heliotrope rash, Gottron’s papules, or Gottron’s sign were not formally documented), the overall clinicopathological pattern—including subsequent treatment response—supported the diagnosis. Comprehensive examinations conducted over a period of 2 years ruled out malignancies (14–16).

The patient was treated with oral prednisone, hydroxychloroquine, and methotrexate for a period of 10 months. During the treatment, there was a gradual reduction in glucocorticoid dosage and liver function, blood counts, and cardiac enzyme levels were monitored on a monthly basis. Unfortunately, as hormones gradually decreased, the rash recurred twice and hormone related side effects appeared. In the 23rd month of treatment, the patient developed a non-healing skin ulcer on his shoulder (**Figure 1C**). Concerned about the repeated rash attacks and hormonal and immunosuppressive side effects, the patient sought alternative treatment options.

## Ozone major blood ozonation treatment process and outcomes

Research has suggested that the optimal ozone concentration for stimulating different cytokines may vary depending on the targeted biological response (17). The ozone concentration of 80 μg/mL used in the present case was selected for two reasons: first, to achieve enhanced immunomodulatory effects targeting the dysregulated inflammatory pathways characteristic of dermatomyositis; and second, to exert sufficient antimicrobial and tissue-repair activity in the context of refractory cutaneous ulceration (18, 19). Although this concentration exceeds the standard range of 60–70 μg/mL recommended in the WFOT São Paulo Document (20) and in the foundational work of Bocci et al. (9), which established that ozone exerts a hormetic dose-response relationship with optimal biological effects within this lower range, the present case involved a refractory condition with active ulceration that may require a stronger oxidative stimulus for immunomodulatory and wound-healing effects. Notably, di Paolo et al. (17) reported the use of concentrations up to 80 μg/mL in extracorporeal ozone blood ozonation for severe refractory infections, providing some precedent for this approach in difficult clinical scenarios. Furthermore, accumulated clinical experience with O_3_-MBO has suggested a generally acceptable safety profile when performed under appropriate monitoring (21). Nevertheless, the use of 80 μg/mL remains above recommended thresholds and should be approached with caution; starting from lower concentrations with stepwise titration is advisable in routine practice. In the present case, no treatment-related adverse events were observed throughout the entire treatment period.

Given that ozone therapy has recently been approved for treating conditions such as psoriasis and Given that ozone therapy has recently been approved for treating conditions such as psoriasis and diabetic foot skin infections (4, 7, 22–24), and considering the patient’s reluctance to continue glucocorticoid therapy due to side effects, we decided to explore its potential benefits for him. Following previously published protocols with minor modifications tailored to the patient’s clinical condition (18, 19), 170 mL of peripheral venous blood was collected via antecubital venous access using a 12-gauge catheter into a sterile disposable ozone-resistant blood collection bag containing sodium citrate anticoagulant. Subsequently, an equal volume (170 mL) of an O_2_/O_3_ gas mixture generated from medical-grade oxygen, with an ozone concentration of 80 μg/mL, was introduced through a sterile antibacterial filter. The blood–gas mixture was gently agitated for several seconds after gas injection.

The procedure involved daily treatment with systemic oxygen–ozone administration for 15 days. Based on the administered gas volume and ozone concentration, the estimated ozone dose per treatment session was approximately 170 mL × 80 μg/mL = 13,600 μg (≈ 13.6 mg). No clinically evident hemolysis, coagulation abnormalities, or acute adverse reactions were observed during treatment. One hour later, he showed an excellent response, and the pain began to subside. After 1 month of treatment, the superficial skin ulcer healed, and the clinical response remained favorable without any adverse effects (**Figure 1C**). So he decided to continue.

In the case presented, the patient added 15 days of O_3_-MBO therapy and 15 days of rest as adjunctive therapy without increasing the hormone dosage. This treatment regimen was repeated cyclically for approximately 15 months in total. This intervention was associated with reductions in serum CK, AST, and ALT, healed the skin ulcers on the shoulders, normalized the skin, reduced the facial edema, as well as alleviation of corticosteroid-related side effects of corticosteroid therapy (**Figure 1D**). These findings suggest that O_3_-MBO may serve as a valuable adjunctive treatment for patients with corticosteroid dependence or refractory dermatological symptoms. The clinical timeline of disease progression and adjunctive O_3_-MBO is summarized in **Figure 1E**.

## Discussion

Treatment of dermatological conditions in patients with dermatomyositis is often challenging. While myositis may respond to treatment with corticosteroids, immunosuppressants, or both, the skin lesions frequently persist (16). Glucocorticoid-induced metabolic complications, such as obesity and edema, remain significant therapeutic challenges. Although leukocyte separation and plasma exchange therapy have been applied in corticosteroid-resistant polymyositis and dermatomyositis, their therapeutic efficacy remains limited, and the safety profile is not well established (25). Furthermore, topical treatments, when used as adjuncts or complementary approaches, are rarely effective in alleviating cutaneous symptoms unless systemic treatments have been successful (26). Although immunoglobulins (IVIg) are approved for use in the treatment of dermatomyositis in adults, they were not attempted in our patient alone (27, 28). Given these shortcomings, we have employed O_3_-MBO as an adjunctive therapeutic measure. In contrast, ozone therapy demonstrated a favorable safety profile and no significant side effects. These findings are also consistent with previous safety evaluations of medical ozone preparations on skin (29).

Numerous studies have confirmed that ozone can facilitate wound healing by improving skin microcirculation, especially in pressure ulcers, diabetic foot, burn wounds, and other ulcers associated with peripheral vascular disease. Notably, ozone promotes platelet aggregation (30) thus implying that ozone could accelerate vascular repair and acute wound healing. Additionally, ozone therapy promotes wound healing via facilitating fibroblast migration (31).

In this case, adjunctive O_3_-MBO therapy was associated with temporal improvements in biochemical parameters and cutaneous lesions. The observed clinical response may be attributable to the combined effects of immunomodulation, attenuation of oxidative stress, and promotion of tissue repair. Importantly, no evidence of hemolysis, coagulation abnormalities, or acute adverse events was observed during treatment. However, oxidative stress biomarkers were not systematically monitored, which represents an important limitation of the present study.

Despite these encouraging observations, several limitations should be acknowledged. First, this is a single-case report, and standardized disease activity assessments, such as the Cutaneous Dermatomyositis Disease Area and Severity Index (CDASI) and Manual Muscle Testing-8 (MMT-8), were not performed; therefore, a causal relationship between ozone therapy and clinical improvement cannot be established. In addition, excessive ozone exposure may induce local oxidative injury. Given the heterogeneity of ozone administration protocols and the lack of long-term safety data, the use of ozone therapy should be approached with caution.

Written informed consent was obtained from the patient for publication of this case report and any accompanying images.

## Data Availability

The original contributions presented in the study are included in the article/supplementary material. Further inquiries can be directed to the corresponding author/s.
